# A comparative study of aesthetic perceptions of malocclusion among general practice dentists, orthodontists and the public using a visual analogue scale (VAS) and the IOTN-AC

**DOI:** 10.4317/jced.53006

**Published:** 2016-12-01

**Authors:** Gonzalo Julián-Castellote, Verónica García-Sanz, José-María Montiel-Company, José-Manuel Almerich-Silla, Carlos Bellot-Arcís

**Affiliations:** 1 Orthodontist. Department of Stomatology, Faculty of Medicine and Dentistry, University of Valencia, Spain; 2 Lecturer on the Master of Orthodontics course, Faculty of Medicine and Dentistry, University of Valencia, Spain; 3 Assistant Lecturer, Department of Stomatology, Faculty of Medicine and Dentistry, University of Valencia, Spain; 4 Tenured Lecturer, Department of Stomatology, Faculty of Medicine and Dentistry, University of Valencia, Spain; 5 Associate lecturer, Department of Stomatology, Faculty of Medicine and Dentistry, University of Valencia, Spain

## Abstract

**Background:**

Perception of malocclusion varies among individuals and among patients and practitioners. Although several indices that tend to coincide in many aspects and unify criteria, no single index has been recognised as the most suitable for assessing orthodontic treatment need. Moreover, orthodontists are not always aware of the differences in perception of malocclusion between patients and practitioners.

**Objetives:**

To examine the perception of dental anaesthetics amongst dentists, orthodontists and the general population, study the relationship between the perception of dental aesthetics and the severity of the malocclusion, using the visual analogue scale and the IOTN-AC, and investigate relationships among the resulting data.

**Study Design:**

Frontal intraoral photographs of 24 cases were classified by the severity of their malocclusion according to the DAI index. The photographs were examined by 150 individuals (30 orthodontists, 30 general dental practitioners and 90 members of the general population), who assessed them on a visual analogue scale and according to the IOTN-AC.

**Results:**

The orthodontists gave the lowest scores on the visual analogue scale, although the differences between the three groups were not significant. For DAI grades 1, 3 and 4, significant differences were found in the IOTN-AC assessments. Here too, the orthodontist group was the most critical.

**Conclusions:**

In general, in all three groups, both the visual analogue scale and IOTN-AC scores increased or decreased in line with the severity of the malocclusion according to the DAI. However, the correlation between these scores was low. The orthodontists scored the malocclusions more critically than the general dentists or the general population with the IOTN-AC, but this difference was not found with the visual analogue scale.

** Key words:**IOTN-AC, DAI, malocclusion.

## Introduction

Malocclusion is difficult to define (1), largely because the perception of this problem varies among individuals and, evidently, among patients and practitioners. Orthodontic patients expect orthodontic treatment to improve their dental and facial aesthetics and, consequently, their popularity and social success (2-5). Orthodontic treatment need is complicated to assess on the basis of studying models or x-rays alone without taking the patient’s perception of his or her problem into account.

No single index has been recognised as the most suitable for assessing orthodontic treatment need (1), although it would appear that a consensus has been reached on the requisites the indices should possess and the occlusal features that should be quantified, as well as on the importance of considering the patient’s own perception of malocclusion. Several indices that tend to coincide in many aspects and unify criteria, and that have already been accepted as valid by various international associations, may be found in the literature. They include the Dental Aesthetic Index (DAI) (6) and the Index of Orthodontic Treatment Need (IOTN) (7).

However, orthodontists are not always aware of the differences in perception between patients and practitioners concerning which features present an occlusion problem or could be improved, or what result defines the success of the treatment. While patients expect results that are defined by social and cultural standards of beauty in their reference group and in society in general, orthodontists prefer to use parameters to reach the diagnosis and plan the subsequent treatment (5,8-10).

Since facial and smile aesthetics are aspects that are highly valued by the population nowadays, and since standards of beauty are not clearly defined, as they depend on social, cultural, individual and professional variables, the aim of this study was to ascertain whether the aesthetic perceptions of the population can be matched to those of dentists and orthodontists and how they relate to the DAI and IOTN-AC indices, in order to make it easier for orthodontists to keep the aesthetic expectations of patients in mind when planning orthodontic treatments.

Consequently, the objectives of this study were to analyse the perception of dental aesthetics among three groups, dentists, orthodontists and the population in general, using a visual analogue scale (VAS) and the aesthetic component of the IOTN (IOTN-AC); to study the relationship between the perception of dental aesthetics and the severity of the malocclusion according to the VAS and the IOTN-AC; and to study the linear correlation between the measurements obtained with the VAS and those of the IOTN-AC.

## Material and Methods

This study was approved by the Human Research Ethics Committee of the University of Valencia (registration number H1396425836238). Before taking measurements from the study models and using the initial photographs from the case records, the patients, parents or tutors were informed and their signed consent and authorisation were obtained.

Out of a total of 280 patients attended by Master of Orthodontics students at the University of Valencia, 24 records were selected. They were classified by the severity of their malocclusions according to the DAI index in order to obtain 6 representative cases for each of the 4 DAI grades. The DAI index is made up of 10 variables: number of visibly missing teeth, crowding in incisal segments, spacing in incisal segments, incisal diastema, largest irregularity in maxilla, largest irregularity in mandible, anterior maxillary overjet, anterior mandibular overjet, vertical anterior openbite and anteroposterior molar relation. Each of these components is multiplied by its regression coefficient and their sum is added to the constant, which has a value of 13, to obtain the DAI score. This places the individual in one of four DAI grades: grade 1, a score of 25 or less, is classed as normal or minor malocclusion; grade 2, with a score of 26 to 30, is considered definite malocclusion, with treatment being elective; grade 3, with a score of 31 to 35, constitutes severe malocclusion, for which treatment is highly desirable; while grade 4, with a score of 36 or more, is very severe or handicapping malocclusion and treatment is mandatory.

The frontal intraoral photographs in maximum intercuspal position from the records of the 24 selected patients were converted from colour to black-and-white with the help of the Adobe Photoshop CC program. The participants were asked to examine each photograph at random for a maximum of 20 seconds and assess it on a visual analogue scale (VAS), where 0 was the worst possible aesthetic perception and 100 the best. The same process was repeated with the IOTN-AC, asking the assessors to indicate which of the 10 photographs on the scale most resembled each of the patients’ photographs.

The study sample was composed of 150 individuals who each rated their aesthetic perception of each of the 24 cases. The 150 individuals belonged to 3 different groups: 30 general dental practitioners, 30 orthodontists and 90 members of the general public. The general dentist group was composed of postgraduate students and lecturers from the Faculty of Medicine and Dentistry of the University of Valencia (Spain). The orthodontist group was made up of practitioners who work exclusively in the field of orthodontics and members of the orthodontics teaching unit of the University of Valencia. The general population group was chosen at random from persons accompanying patients to the University of Valencia dental clinic. They were asked whether they would volunteer to take part in the study. All accepted and signed an informed consent form.

The data from the forms were stored in a spreadsheet, using the Microsoft® *Excel 2011®* program. The statistical analysis was performed with IBM® *SPSS statistics v 21.0®* software.

## Results

The mean age of the sample was 42.9 years and the distribution by gender was very balanced (50% men and 50% women).  Table 1 shows the distribution of the three groups in the sample by gender and age.

**Table 1:**
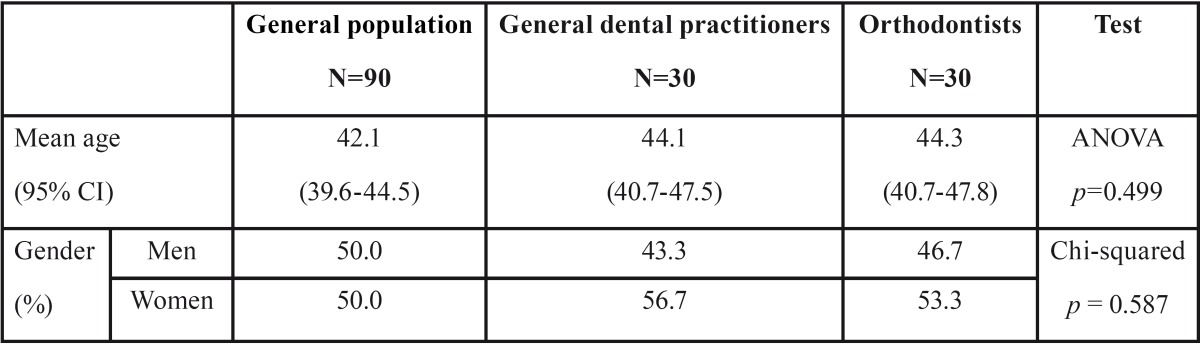
Distribution of the sample by age and gender.

**Figure 1:**
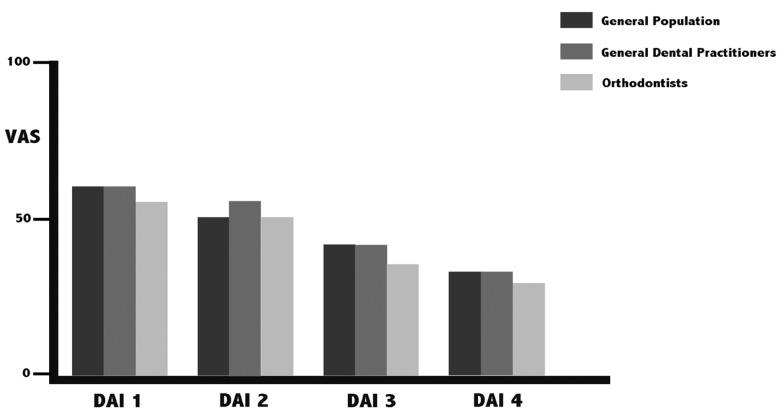
Mean VAS scores of the three groups by DAI grade.

**Figure 2:**
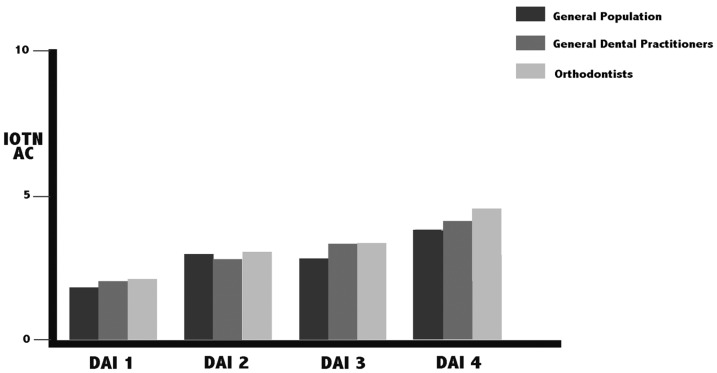
Mean IOTN-AC scores of the three groups by DAI grade.

On comparing the mean VAS scores of each group, no significant inter-group differences were found in any of the DAI grades, although the orthodontists gave the lowest scores in all four grades. Figure 1 shows that as the DAI grade rose, the VAS score fell. With the IOTN-AC, the three groups differed significantly in DAI grades 1, 3 and 4, with the orthodontist group again being the most severe ( Table 2). As the DAI grade rose, so did the IOTN-AC scores (Fig. 2).

**Table 2:**
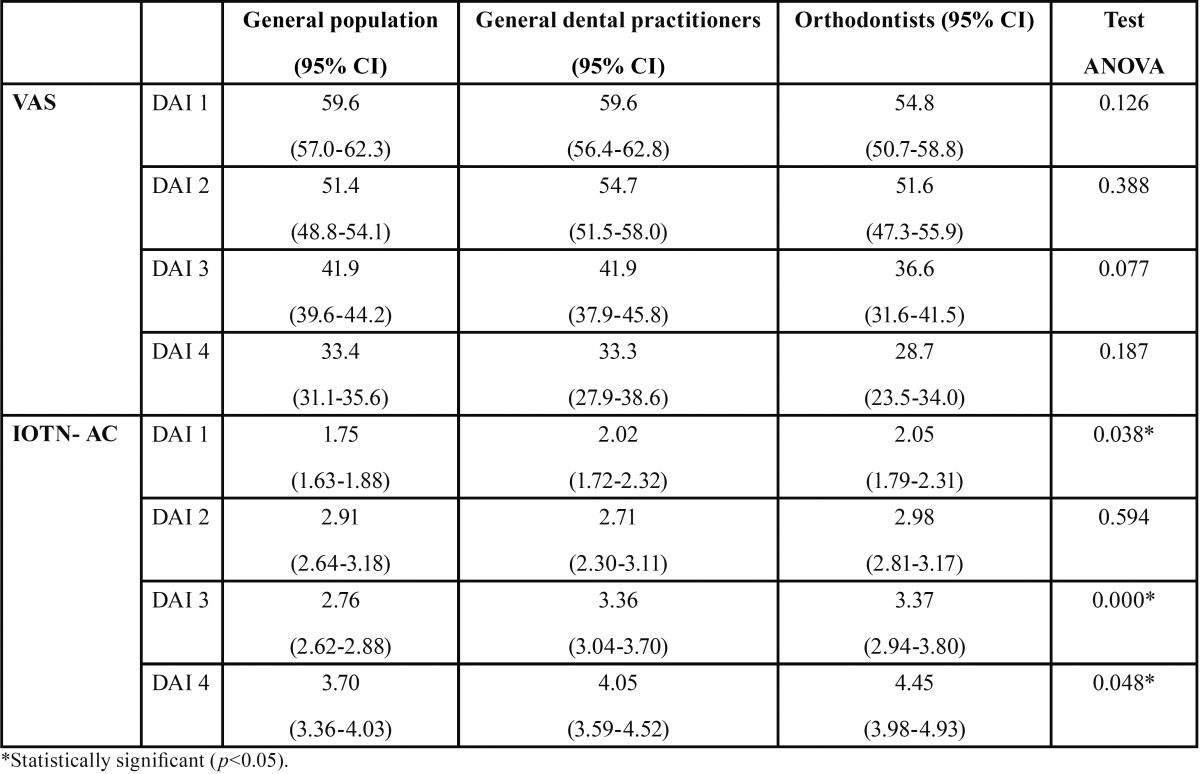
Distribution of the sample by age and gender.

 Table 3 shows the Pearson’s correlation coefficients on comparing the VAS and IOTN-AC scores of the three groups (general population, general dental practitioners and orthodontists). These coefficients were obviously negative, although their values were low and not statistically significant. The coefficients of the orthodontist group were higher than those of the general public and the general practice dentists.

**Table 3:**
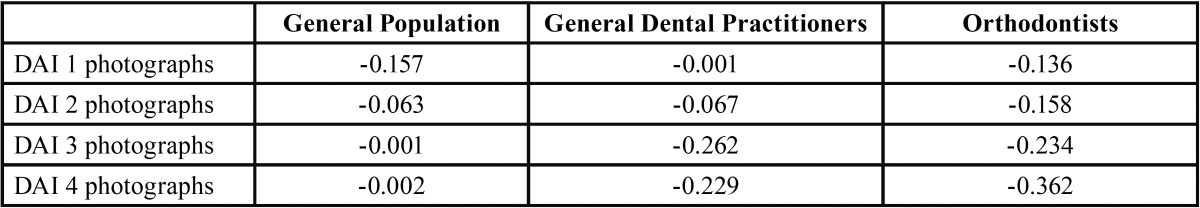
Pearson’s correlation coefficient between VAS and IOTN-AC scores for the different groups of photographs (DAI 1, 2, 3 and 4).

## Discussion

Measuring the aesthetic impairment that a particular malocclusion can cause and the degree to which this affects the social relations of the patient is not a simple task, although valid questionnaires have been designed for this purpose (11).

The objective of this study was solely to compare the aesthetic perceptions of the patients, expressed on a visual analogue scale and through the aesthetic component of the IOTN (methods that have been used for many years), with those of orthodontists, as the specialists responsible for treating malocclusions, and of general dental practitioners, as the referrers, the intermediate step or link between patients and orthodontists.

The reason for using two different scales to measure aesthetic perception is that individuals may have difficulty in identifying malocclusions with one of the 10 standard photographs of the IOTN-AC, none of which show openbite or anterior crossbite (12).

The 24 cases selected (6 for each of the 4 DAI grades) showed a wide range of the malocclusions present in the population. However, it should be mentioned that occlusive components such as overjet, which is quite heavily weighted in the DAI index calculation, can be difficult to assess from frontal photographs. All the frontal intraoral photographs were taken in maximum intercuspal position and with labial retraction so that the aesthetic perception of the position of the anterior teeth could be assessed in the same way as in the IOTN-AC. To avoid any bias in aesthetic perception due to other factors such as variations in tooth colour, periodontal problems or lack of hygiene, the colour photographs were converted to black and white with the help of the Adobe Photoshop CC program.

The Pearson’s correlation coefficients between general practice dentists, orthodontists and the general population were low and not statistically significant, although the orthodontists were always stricter in their assessment of the aesthetics of the malocclusion. Flores-Mir *et al.* obtained similar results although the correlation between the three scales they compared (IOTN-AC, OASIS and VAS) was moderate, unlike the low coefficients obtained in the present study (13). However, Murakami *et al.* encountered significant correlations when using the PAR index and a VAS in a study that compared the perception of dentistry students and orthodontists (14).

Another study by Hamamci *et al.* investigated patients’ perceptions of their malocclusions and their treatment need as measured by the DAI, concluding that the correlation between these was weak (15). The present study observed a clear linear trend on plotting the aesthetic perception score against the severity of the case. Within each group, the VAS scores fell as the DAI grade rose. The same occurred with the IOTN-AC score, but in the opposite direction, increasing as the malocclusion worsened.

On calculating the mean IOTN-AC scores assigned to each DAI grade and comparing the results of the three groups, the differences between orthodontists, dentists and the general population were significant in all DAI grades except 2. These results agree with those of most authors (16-18), who have observed a tendency for clinicians to be more critical than the general public.

In the present study, the orthodontists tended to be much more critical than the other two groups. This agrees with the results of Murakami *et al.*, who found no significant differences between students and orthodontists in cases with low treatment need, but differences in cases with a PAR greater than 23, where the orthodontists perceived a greater treatment need (14).

One of the main limitations of the study is sample size, although the difficulty of encountering 30 practitioners working exclusively in the field of orthodontics should be taken into account.

## Conclusions

- In general, both the visual analogue scale scores and the IOTN-AC scores increased or decreased in line with the severity of the malocclusion according to the DAI in all three study groups. However, the correlation between these scores was low.

- With the IOTN-AC, the orthodontists scored the malocclusions more severely than the general practice dentists or the general population, but did not exhibit the same difference in the visual analogue scale scores.

## References

[1] Bellot-Arcís C, Montiel-Company JM, Almerich-Silla JM, Paredes-Gallardo V, Gandía-Franco JL (2012). The use of occlusal indices in high-impact literature. Community Dent Health.

[2] Shaw WC, Rees G, Dawe M, Charles CR (1985). The influence of dentofacial appearance on the social attractiveness of young adults. American Journal of Orthodontics.

[3] Kerosuo H, Hausen H, Laine T, Shaw WC (1995). The influence of incisal maloclusion on the social attractiveness of young adults in Finland. European Journal of Orthodontics.

[4] Birkeland K, Bøe OE, Wisth PJ (2000). Relationship between occlusion and satisfaction with dental appearance in orthodontically treated and untreated groups. A longitudinal study. European Journal of Orthodontics.

[5] Kiekens RMA, Maltha JC, van ´t Hof MA, Kuijpers-Jagtman AM (2006). Objective measures as indicators for facial esthetics in white adolescents. Angle Orthodontist.

[6] Cons NC, Jenny J, Kohout FJ (1986). DAI The Dental Aesthetic Index Iowa City, Iowa.. College of Dentistry. University of Iowa.

[7] Brook PH, Shaw WC (1989). The development of an index of orthodontic treatment priority. Eur J Orthod.

[8] Spyropoulos MN, Halazonetis DJ (2001). Significance of the soft tissue profile on facial esthetics. American Journal of Orthodontics and Dentofacial Orthopedics.

[9] McKoy-White J, Evans CA, Viana G, Anderson NK, Giddon DB (2006). Facial profile preferences of black women before and after orthodontic treatment. American Journal of Orthodontics and Dentofacial Orthopedics.

[10] Miner RM, Anderson NK, Evans CA, Giddon DB (2007). The perception of children's computer-imaged facial profiles by patients, mothers and clinicians. Angle Orthodontist.

[11] Bellot-Arcís C, Montiel-Company JM, Almerich-Silla JM (2013). Psychosocial impact of malocclusion in Spanish adolescents. The Korean Journal of Orthodontics.

[12] Bellot-Arcís C, Montiel-Company JM, Pinho T, Almerich-Silla JM (2015). Relationship between perception of malocclusion and the psychological impact of dental aesthetics in university students. J. Clin Exp Dent.

[13] Flores-Mir C, Major PW, Salazar FR (2004). Self-perceived orthodontic treatment need evaluated through 3 scales in a university population. J Orthod.

[14] Murakami T, Fujii A, Kawabata Y, Takakura H, Yamaue R, Balam TA (2013). Relationship between orthodontic expertise and perception of need for orthodontic treatment for mandibular protrusion in Japan. Acta Med Okayama.

[15] Hamamci N, Basaran G, Uysal E (2009). Dental Aesthetic Index scores and perception of personal dental appearance among Turkish university students. European Journal of Orthodontics.

[16] Shaw WC, Lewis HG, Robertson NRE (1975). Perception of malocclusion. British Dental Journal.

[17] Lindsay S, Hodgkins J (1983). Children's perceptions of their own malocclusions. British Journal of Orthodontics.

[18] Mandall NA, Wright J, Conboy FM, O'Brien KD (2001). The relationship between normative orthodontic treatment need and measures of consumer perception. Community Dental Health.

